# Supplementary material for: Probing material absorption and optical nonlinearity of integrated photonic materials
Source: Nat Commun. 2022 Jun 9;13:3323. doi: 10.1038/s41467-022-30966-5 (PMC9184588; doi:10.1038/s41467-022-30966-5)
Supplement: Supplementary file 1 — Supplementary Information [file 41467_2022_30966_MOESM1_ESM.pdf]

Supplementary information: Probing material absorption and optical nonlinearity of integrated photonic materials

Maodong Gao[1,*], Qi-Fan Yang[1,*], Qing-Xin Ji[1,*], Heming Wang[1], Lue Wu[1], Boqiang Shen[1], Junqiu Liu[2], Guanhao Huang[2], Lin Chang[3], Weiqiang Xie[3], Su-Peng Yu[4], Scott B. Papp[4,†], John E. Bowers[3,†], Tobias J. Kippenberg[2,†] and Kerry J. Vahala[1,†]

[1]T. J. Watson Laboratory of Applied Physics, California Institute of Technology, Pasadena, California 91125, USA

[2]Institute of Physics, Swiss Federal Institute of Technology Lausanne (EPFL), CH-1015 Lausanne, Switzerland

[3]ECE Department, University of California Santa Barbara, Santa Barbara, CA 93106, USA

[4]National Institute of Standards and Technology, Boulder, CO 80305, USA

[*]These authors contributed equally to this work.

[†]Corresponding author: scott.papp@nist.gov, jbowers@ucsb.edu, tobias.kippenberg@epfl.ch, vahala@caltech.edu

CONTENTS

I. Supplementary Note 1: Resonance shifts induced by nonlinearity and absorption 2
A. Resonant frequency under refractive index perturbation 2
B. Impact of Kerr nonlinearity 3
C. Impact of linear absorption 3
D. Calibration of resonance thermal shift 5

II. Supplementary Note 2: Model of the sum and ratio experiments 5
A. Lineshape with a Fabry–Pérot background 5
B. Model of the ratio experiment 8
C. Sources of systematic errors in Q_{abs} and n_2 9

III. Supplementary Note 3: Detailed measurement results 10
A. Measurement of SiO_2 microresonators 10
B. Measurement of Si_3N_4 microresonators 11
C. Measurement of $Al_{0.2}Ga_{0.8}As$ microresonators 13
D. Measurement of Ta_2O_5 microresonators 14

IV. Supplementary Note 4: Miller's rule for nonlinear susceptibility 15

References 16

I. SUPPLEMENTARY NOTE 1: RESONANCE SHIFTS INDUCED BY NONLINEARITY AND ABSORPTION

A. Resonant frequency under refractive index perturbation

Here we present the *ab initio* derivation of linear and nonlinear parameters of a circular microresonator consisting of heterogeneous structures with spatially-varying refractive index $n_o(r,\theta,z)$ and Kerr coefficient $n_2(r,\theta,z)$, where (r,θ,z) is the cylindrical coordinate. We start with the Helmholtz equation for a single-mode within the resonator,

$$-\nabla\times\nabla\times\tilde{\mathbf{E}}(\mathbf{r},\omega)+\frac{n_o^2\omega^2}{c^2}\tilde{\mathbf{E}}(\mathbf{r},\omega_0)=0, \tag{S1}$$

where $\tilde{\mathbf{E}}$ is the (real) modal electric field, c is the vacuum speed of light, and ω is the resonance angular frequency. For convenience, $\tilde{\mathbf{E}}$ can be expressed as a phasor,

$$\tilde{\mathbf{E}}=\frac{1}{2}\mathbf{E}+\text{c.c.},\quad \mathbf{E}=a\mathbf{F}(r,\theta,z)e^{-i\omega t}, \tag{S2}$$

where a is the complex mode amplitude and $\mathbf{F}$ is the field distribution. For $|a|^2$ to represent the optical energy stored in the mode, the normalization for $\mathbf{F}$ reads

$$\frac{\varepsilon_0}{2}\int n_o^2|\mathbf{F}|^2\mathrm{d}V=1, \tag{S3}$$

where $|\mathbf{F}|^2\equiv\mathbf{F}\cdot\mathbf{F}^*$ and the volume integral goes over the entire resonator. Here we assume that the modal field is sufficiently localized so convergence problems associated with quasimodes can be neglected.

For the Helmholtz equation Eq. (S1), ω^2 can be expressed in its variational form:

$$\omega^2=c^2\frac{\int|\nabla\times\mathbf{F}|^2\mathrm{d}V}{\int n_o^2|\mathbf{F}|^2\mathrm{d}V}. \tag{S4}$$

For a slight change in refractive index Δn that is applied externally, the perturbed eigenfrequency can be written similarly as:

$$(\omega_0+\delta\omega_0)^2=c^2\frac{\int|\nabla\times\mathbf{F}|^2\mathrm{d}V}{\int(n_o'+\Delta n)^2|\mathbf{F}|^2\mathrm{d}V}, \tag{S5}$$

where the field $\mathbf{F}$ can be regarded as not changing up to first order of Δn, and n_o' is the refractive index at the new eigenfrequency $\omega_0+\delta\omega_0$ to take account of the chromatic dispersion of the material. n_o' can be expanded as

$$n_o'\approx n_o+\frac{\partial n_o}{\partial\omega_0}\delta\omega_0=n_o+(n_g-n_o)\frac{\delta\omega_0}{\omega_0}, \tag{S6}$$

where n_g is the group index associated with n_o. By expanding Eq. (S5) up to first order in Δn, we can obtain

$$\begin{aligned}\frac{1}{(\omega_0+\delta\omega_0)^2}&\approx\frac{\int n_o^2|\mathbf{F}|^2\mathrm{d}V}{c^2\int|\nabla\times\mathbf{F}|^2\mathrm{d}V}+\frac{2\int[n_o(n_g-n_o)\delta\omega_0/\omega_0]\,|\mathbf{F}|^2\mathrm{d}V}{c^2\int|\nabla\times\mathbf{F}|^2\mathrm{d}V}+\frac{2\int n_o\Delta n|\mathbf{F}|^2\mathrm{d}V}{c^2\int|\nabla\times\mathbf{F}|^2\mathrm{d}V}\\&=\frac{1}{\omega_0^2}-\frac{2}{\omega_0^2}\frac{\delta\omega_0}{\omega_0}+2\frac{\delta\omega_0}{\omega_0}\frac{\int n_on_g|\mathbf{F}|^2\mathrm{d}V}{c^2\int|\nabla\times\mathbf{F}|^2\mathrm{d}V}+\frac{2\int n_o\Delta n|\mathbf{F}|^2\mathrm{d}V}{c^2\int|\nabla\times\mathbf{F}|^2\mathrm{d}V}\\&\approx\frac{1}{(\omega_0+\delta\omega_0)^2}+\frac{2}{c^2\int|\nabla\times\mathbf{F}|^2\mathrm{d}V}\left(\frac{\delta\omega_0}{\omega_0}\int n_on_g|\mathbf{F}|^2\mathrm{d}V+\int n_o\Delta n|\mathbf{F}|^2\mathrm{d}V\right)\end{aligned} \tag{S7}$$

Therefore the $\delta\omega_0$ can be solved as,

$$\frac{\delta\omega_0}{\omega_0}=-\frac{\int n_o\Delta n|\mathbf{F}|^2\mathrm{d}V}{\int n_on_g|\mathbf{F}|^2\mathrm{d}V}. \tag{S8}$$

B. Impact of Kerr nonlinearity

The local refractive change induced by the Kerr nonlinearity for an isotropic material has the form

$$\Delta n = \frac{\varepsilon_o c n_o n_2}{2}|\mathbf{E}|^2, \tag{S9}$$

where n_2 is the nonlinear index associated with n_o. Substituting Δn into Eq. (S8), we have

$$\frac{\delta\omega_0}{\omega_0} = -\frac{\varepsilon_o c}{2}|a|^2 \frac{\int n_o^2 n_2 |\mathbf{F}|^4 \mathrm{d}V}{\int n_o n_g |\mathbf{F}|^2 \mathrm{d}V}. \tag{S10}$$

To gain insight into the expression, we define the weighted nonlinear index,

$$\overline{n_2} = \frac{\int n_o^2 n_2 |\mathbf{F}|^4 \mathrm{d}V}{\int n_o^2 |\mathbf{F}|^4 \mathrm{d}V}, \tag{S11}$$

the effective mode volume,

$$V_{\text{eff}} = \frac{\int n_o^2 |\mathbf{F}|^2 \mathrm{d}V \int |\mathbf{F}|^2 \mathrm{d}V}{\int n_o^2 |\mathbf{F}|^4 \mathrm{d}V}, \tag{S12}$$

and the weighted index product,

$$\overline{n_o n_g} = \frac{\int n_o n_g |\mathbf{F}|^2 \mathrm{d}V}{\int |\mathbf{F}|^2 \mathrm{d}V}. \tag{S13}$$

Using these relations, the eigenfrequency shift can be expressed as

$$\delta\omega_0 = -\frac{\omega_0 c \overline{n_2}}{\overline{n_o n_g} V_{\text{eff}}}|a|^2 = -\frac{\omega_0 c \overline{n_2}}{\overline{n_o n_g}}\rho, \tag{S14}$$

where we have used the normalization for $\mathbf{F}$ in Eq. (S3), and $\rho = |a|^2/V_{\text{eff}}$ is the intracavity energy density as in the main text. Therefore, the nonlinear coupling coefficient g, as defined in Eq. (1) in the main text, takes the simple form

$$g = \frac{\omega c \overline{n_2}}{\overline{n_o n_g}}, \tag{S15}$$

which is the g expression in Eq. (2) in the main text. We note that, for resonators made with a single material, the averages in the expression drop out, and we recover the conventional result $g = \omega c n_2/(n_o n_g)$.

C. Impact of linear absorption

The local refractive change induced by temperature change reads

$$\Delta n = n_o \alpha_n \delta T, \tag{S16}$$

where $\alpha_n = (\delta n_o/\delta T)/n_o$ is the thermorefractive coefficient associated with n_o and δT is the temperature field. Substituting Δn into Eq. (S8), we have

$$\frac{\delta\omega_0}{\omega_0} = -\frac{\int n_o^2 \alpha_n \delta T |\mathbf{F}|^2 \mathrm{d}V}{\int n_o n_g |\mathbf{F}|^2 \mathrm{d}V}. \tag{S17}$$

For later convenience, we define a temperature average with respect to the optical field,

$$\overline{\delta T} = \frac{\int n_o^2 \alpha_n \delta T |\mathbf{F}|^2 \mathrm{d}V}{\int n_o^2 \alpha_n |\mathbf{F}|^2 \mathrm{d}V} \tag{S18}$$

such that different temperature distributions with equal $\overline{T}$ will induce the same frequency shift:

$$\frac{\delta\omega_0}{\omega_0} = -\overline{\delta T}\frac{\int n_o^2\alpha_n|\mathbf{F}|^2\mathrm{d}V}{\int n_o n_g|\mathbf{F}|^2\mathrm{d}V}. \tag{S19}$$

The proportional coefficient $\delta\omega_0/\overline{\delta T}$ is measured in the experiment through the resonance thermal shift. However, the measurement does not distinguish thermo-refractive and thermo-elastic effects, and requires correction (see Section ID).

The thermal response properties of the microresonators are evaluated with finite-element method simulations (COMSOL Multiphysics). The constants of the materials used in the simulations are shown in Table. S1.

Material	Density ρ_m (kg·m^{-3})	Thermal conductivity k (W·m^{-1}·K^{-1})	Heat capacity C (J·kg^{-1}·K^{-1})
Si (ref.[1])	2.33×10^3	130	700
SiO_2 (ref.[1])	2.2×10^3	1.4	740
Si_3N_4 (ref.[1,2])	3.17×10^3	30	800
$Al_xGa_{1-x}As$ (ref.[3])	$(5.32-1.56x)\times10^3$	$55-212x+248x^2$	$320+132x$
Ta_2O_5	6.85×10^3 (ref.[4])	0.4 (ref.[5])	306 (ref.[6])

TABLE S1: **Thermal constants of resonator materials used in finite-element method simulations.** The thermal conductivity of Ta_2O_5 is further discussed in Section IIID.

To obtain the δT field and the thermal diffusion responsivity, the electrical field distribution $\mathbf{F}$ is simulated and used as the heat source of the thermal diffusion equation:

$$\rho_\mathrm{m}C\frac{\partial T}{\partial t} - k\nabla^2 T = \frac{1}{2}\varepsilon_o n_o^2|a|^2\kappa_a|\mathbf{F}|^2, \tag{S20}$$

where the right hand side represents the power density absorbed, and $|a|^2$ is intracavity energy. Applying time domain Fourier transform gives

$$(i\Omega\rho_\mathrm{m}C - k\nabla^2)\widetilde{T}(\Omega) = \frac{1}{2}\varepsilon_o n_o^2\widetilde{|a|^2}(\Omega)\kappa_a|\mathbf{F}|^2, \tag{S21}$$

where Ω is the modulation frequency, $\widetilde{T}(\Omega)$ is the Fourier transform of the temperature T and $\widetilde{|a|^2}(\Omega)$ is amplitude of the modulation. For the sum measurement, steady-state index changes are used, where $\delta T = \widetilde{T}(\Omega=0)$, to calculate factor $\overline{\delta T}/P_\mathrm{abs}$ in Eq. (2) in the main text. The ratio experiment requires the dynamical behavior of T, and we define a thermal response function $\widetilde{r}(\Omega)$ as

$$\widetilde{r}(\Omega) = \frac{\widetilde{T}(\Omega)}{\widetilde{|a|^2}(\Omega)}\Bigg/\frac{\widetilde{T}(\Omega=0)}{\widetilde{|a|^2}(\Omega=0)}. \tag{S22}$$

The simulation result of $\overline{\delta T}/P_\mathrm{abs}$ and $\widetilde{r}(\Omega)$ for each material is presented in Supplementary note 3.

In addition, the rate of total power loss inducing the temperature change equals

$$P_\mathrm{abs} = \frac{1}{2}\varepsilon_o|a|^2\int\kappa_a n_o^2|\mathbf{F}|^2\mathrm{d}V \tag{S23}$$

and can be written as $P_\mathrm{abs} = \overline{\kappa_\mathrm{a}}|a|^2$, where the averaged absorption rate reads (Normalization relation in Eq. (S3) is used)

$$\overline{\kappa_\mathrm{a}} = \frac{P_\mathrm{abs}}{|a|^2} = \frac{\int\kappa_\mathrm{a}n_o^2|\mathbf{F}|^2\mathrm{d}V}{\int n_o^2|\mathbf{F}|^2\mathrm{d}V}. \tag{S24}$$

Now the absorption can be related to cavity resonance shift by

$$\delta\omega_0 = \left(\frac{\delta\omega_0}{\overline{\delta T}}\right)\left(\frac{\overline{\delta T}}{P_\mathrm{abs}}\right)P_\mathrm{abs} = \left(\frac{\delta\omega_0}{\overline{\delta T}}\right)\left(\frac{\overline{\delta T}}{P_\mathrm{abs}}\right)\overline{\kappa_\mathrm{a}}|a|^2, \tag{S25}$$

where the first coefficient comes from experiment (see Fig. 2d in the main text) and the second coefficient comes from thermal simulation as described above. Using $\delta\omega_0 = -\alpha|a|^2/V_{\text{eff}}$ (as defined in Eq. (1) in the main text with $\rho \equiv |a|^2/V_{\text{eff}}$), α can be calculated as

$$\alpha = -\overline{\kappa_{\text{a}}} \left(\frac{\delta\omega_0}{\overline{\delta T}}\right) \left(\frac{\overline{\delta T}}{P_{\text{abs}}}\right) V_{\text{eff}}, \tag{S26}$$

which is the α expression in Eq. (2) in the main text.

D. Calibration of resonance thermal shift

In the sum experiment, resonance shift originates from localized material absorption. This increases the resonator temperature locally, and shifts the resonance primarily through the thermo-optical effect (factor α_n is defined in Eq. (S16)). However, when the resonance shift is calibrated using a thermoelectric cooler (TEC) (see Fig. 2d in the main text), the entire chip is heated, and the resonance shift therefore includes a contribution from the thermo-elastic expansion created by the silicon substrate. To remove this extra contribution, the coefficient of linear thermal expansion $\alpha_{l,\text{sub}}$ of the silicon substrate is subtracted. As a check of this approach, Table S2 compiles measured values for $\alpha_n + \alpha_{l,\text{sub}}$ using the TEC as described in the main text along with values for α_n and α_l (coefficient of linear thermal expansion) reported in the literature for the relevant materials. The TEC measured values are close compared to the reported α_n with the silicon (substrate material) α_l added. The discrepancy between α_n reported for $Al_{0.2}Ga_{0.8}As$ makes the same comparison difficult, but the result for this case falls reasonably close to the measured value.

10^{-5} K^{-1}	SiO_2	Si_3N_4	$Al_{0.2}Ga_{0.8}As$	Ta_2O_5	Si (Substrate)
α_n, reported	0.752 (ref.[7])	1.25 (ref.[8])	6.970 (ref.[9])<br>6.054 (ref.[11])	0.29 (ref.[10])	–
α_l, reported	0.055 (ref.[12])	0.33 (ref.[13])	0.562 (ref.[3])	0.24 (ref.[14])	0.26 (ref.[15])
$\alpha_n + \alpha_{l,\text{sub}}$, measured (TEC)	0.95	1.47	6.798	0.52	–

TABLE S2: **Thermo-optic and thermo-elastic coefficients reported in the literature compared with the measured combined effect determined by heating of the resonator chip.**

II. SUPPLEMENTARY NOTE 2: MODEL OF THE SUM AND RATIO EXPERIMENTS

A. Lineshape with a Fabry–Pérot background

Three of the devices being tested (Si_3N_4, $Al_{0.2}Ga_{0.8}As$ and Ta_2O_5) feature integrated waveguides, so that light is coupled to the chip through the waveguide facets located at the side of the chips. Of these three devices, the facets of the $Al_{0.2}Ga_{0.8}As$ are angled, while the Si_3N_4 and Ta_2O_5 have flat facets, causing the light in the waveguide to be reflected at these endpoints. The reflections form a Fabry–Pérot (FP) cavity out of the on-chip waveguide, and the measured resonator transmission lineshapes appear with a modulated background. In the following we model this FP background in order to extract resonator properties accurately.

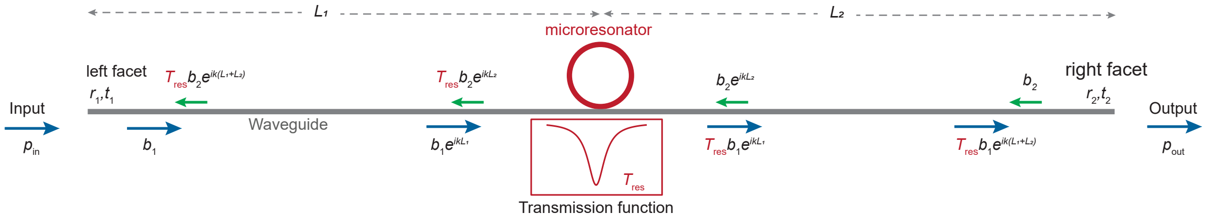

FIG. S1: **Schematic used for modelling transmission spectra with FP backgrounds.** The micro-resonator (red ring) is coupled to the bus waveguide (gray line). Quantities are defined in the text.

We denote b_1 (b_2) as the slowly-varying field just inside the input (output) waveguide facet propagating towards the resonator (Fig. S1). At the output facet, the field propagating towards the facet reads $T_{\mathrm{res}}\mathrm{e}^{ik(L_1+L_2)}b_1$, where T_{res} is the linear transmission function of the resonator, k is the wavevector, and L_1 and L_2 are waveguide lengths from the resonator coupling point to the input and output facets, respectively. This expression assumes no loss on the waveguide, and the exponential factor accounts for the propagation phase. Similarly, at the input facet, the field propagating towards the facet reads $T_{\mathrm{res}}\mathrm{e}^{ik(L_1+L_2)}b_2$. Reflection at the facets cause the fields in the two directions to couple together as follows:

$$b_1 = r_1 T_{\mathrm{res}} b_2 \mathrm{e}^{ik(L_1+L_2)} + t_1 p_{\mathrm{in}}, \tag{S27}$$

$$b_2 = r_2 T_{\mathrm{res}} b_1 \mathrm{e}^{ik(L_1+L_2)}, \tag{S28}$$

where r_1 (r_2) and t_1 (t_2) are reflection and transmission coefficients, respectively, at the input (output) waveguide facet, and p_{in} is the input field at the waveguide facet. Solving the above equations gives

$$b_1 = \frac{t_1 p_{\mathrm{in}}}{1 - r_1 r_2 T_{\mathrm{res}}^2 \mathrm{e}^{2ikL_{\mathrm{wg}}}}. \tag{S29}$$

with $L_{\mathrm{wg}} \equiv L_1 + L_2$. The output field from the waveguide p_{out} is

$$p_{\mathrm{out}} = t_2 T_{\mathrm{res}} b_1 \mathrm{e}^{ikL_{\mathrm{wg}}}. \tag{S30}$$

and the output power can be found as

$$P_{\mathrm{out}} = |p_{\mathrm{out}}|^2 = |t_1 t_2|^2 \frac{|T_{\mathrm{res}}|^2}{|1 - r_1 r_2 T_{\mathrm{res}}^2 \mathrm{e}^{2ikL_{\mathrm{wg}}}|^2} |p_{\mathrm{in}}|^2. \tag{S31}$$

To see how this power transmission gives the FP background, we consider the case when the resonator is not on resonance, i.e. $T_{\mathrm{res}} = 1$. The above equation simplifies to

$$P_{\mathrm{out}} = \frac{|t_1 t_2|^2}{|1 - r_1 r_2 \mathrm{e}^{2ikL_{\mathrm{wg}}}|^2} |p_{\mathrm{in}}|^2. \tag{S32}$$

For later convenience, the wavevector k is replaced with the cavity detuning of a certain resonance, $\Delta_{\mathrm{res}} = \omega_{\mathrm{c,res}} - \omega_p$, where $\omega_{\mathrm{c,res}}$ is the resonance frequency and ω_p is the pump frequency. The exponential factor can then be written as,

$$\exp(2ikL_{\mathrm{wg}}) = \exp\left[2i\frac{L_{\mathrm{wg}}}{c}(\omega_{\mathrm{c,res}} - \Delta_{\mathrm{res}})\right] = \exp\left(-i\frac{\Delta_{\mathrm{res}}}{\omega_{\mathrm{FP}}} + i\phi\right) \tag{S33}$$

where we have identified $\omega_{\mathrm{FP}} = c/(2L_{\mathrm{wg}})$ as the free spectral range of the FP cavity and $\phi = \omega_{\mathrm{c,res}}/\omega_{\mathrm{FP}}$ is a phase offset. Therefore, the power transmission reads

$$P_{\mathrm{out}} = \frac{|t_1 t_2|^2}{|1 - r_1 r_2 \mathrm{e}^{-i\Delta_{\mathrm{res}}/\omega_{\mathrm{FP}} + i\phi}|^2} |p_{\mathrm{in}}|^2. \tag{S34}$$

From here, the total reflection $r = |r_1 r_2|$, total transmission $|t_1 t_2|$ and the phase offset ϕ can be fitted from the experiment data. We note that the phase of $r_1 r_2$ can be absorbed into ϕ and the fitting parameters can be restricted to be real.

Near the resonance frequency, the linear transmission T_{res} can be found from the coupled-mode equation and the input-output relations of the resonator:

$$\frac{\mathrm{d}a_{\mathrm{res}}}{\mathrm{d}t} = -\left(\frac{\kappa_{\mathrm{res}}}{2} + i\Delta_{\mathrm{res}}\right) a_{\mathrm{res}} + \sqrt{\kappa_{e,\mathrm{res}}} a_{\mathrm{in}}, \tag{S35}$$

$$a_{\mathrm{out}} = -\sqrt{\kappa_{e,\mathrm{res}}} a_{\mathrm{res}} + a_{\mathrm{in}}, \tag{S36}$$

where a_{res} is the field amplitude of the resonance, κ_{res} and $\kappa_{e,\mathrm{res}}$ are the intrinsic loss rate and external coupling rate of the resonance mode respectively, a_{in} and a_{out} are the waveguide fields before and after the resonator, and Δ_{res} is

the detuning introduced previously. At steady state ($\mathrm{d}a_{\mathrm{res}}/\mathrm{d}t = 0$) the transmission can be solved as

$$T_{\mathrm{res}} = \frac{a_{\mathrm{out}}}{a_{\mathrm{in}}} = 1 - \frac{\kappa_{e,\mathrm{res}}}{\kappa_{\mathrm{res}}/2 + i\Delta_{\mathrm{res}}}. \tag{S37}$$

The overall power transmission now reads

$$P_{\mathrm{out}} = |t_1 t_2|^2 \frac{|T_{\mathrm{res}}|^2}{|1 - r_1 r_2 T_{\mathrm{res}}^2 \mathrm{e}^{-i\Delta_{\mathrm{res}}/\omega_{\mathrm{FP}} + i\phi}|^2} |p_{\mathrm{in}}|^2. \tag{S38}$$

where T_{res} should be substituted with the previous equation, and is Eq. (5) in the main text. From here, κ_{res} and $\kappa_{e,\mathrm{res}}$ can be fitted using experimental data and parameters from the previous stage.

It is important to note that the lineshape is not a product of the FP background and the bare lorentzian resonance $|T_{\mathrm{res}}|^2$. The appearance of T_{res} on the denominator of P_{out} creates interference between the two lineshapes, which leads to about 20% error for Q values for the current data unless the FP interference is taken into account. An example of a Ta_2O_5 resonance is shown in Fig. S2.

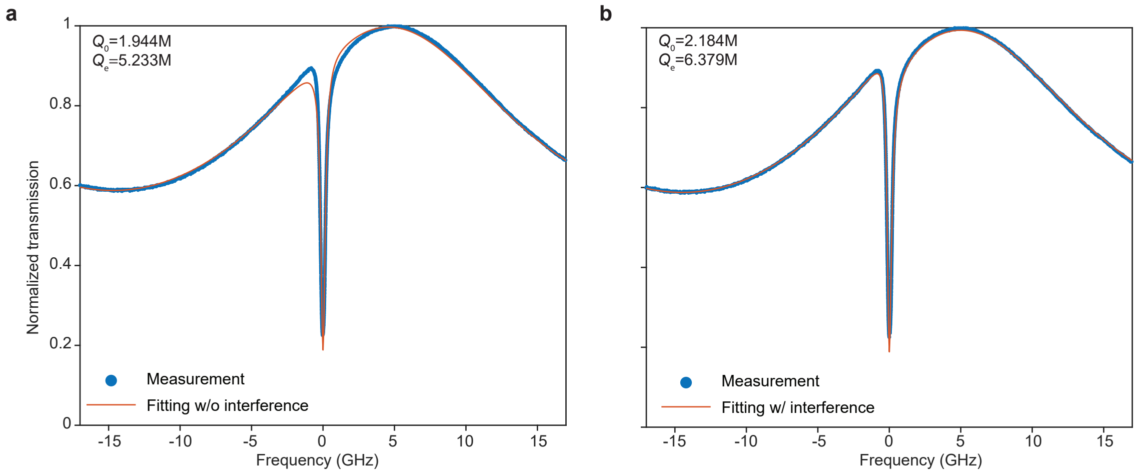

FIG. S2: Q **fitting with FP background.** **a** and **b** are the results of fitting the same resonance of a Ta_2O_5 resonator TE mode at $1559.4nm$. **a.** An overly simplified model of the FP background times the Lorentzian resonance is used here to fit the experiment data. A deviation between data and fitting can be observed around the resonance. **b.** The improved model (Eq. (S38) and Eq. (S37)) is used to fit data and gives a more accurate fitting. Q_0 is the fitted intrinsic Q and Q_e is the fitted external (coupling) Q. The Q factors obtained in both cases have a difference about 20%, showing that it is essential to take the FP interference effect into account.

The above results do not apply to the case when the resonance undergoes nonlinear broadening, where T_{res} now depends on the pump power at the resonator. The analysis is further complicated by the fact that the transmitted power after the resonator will be reflected to affect the pumping power, and the backward-propagating light sees a different resonance in the resonator due to the difference between self- and cross-phase Kerr modulations. To get around these problems, we assume that at all frequencies, the pumping power for the resonator is the waveguide power with FP effects but without the cavity resonance, and we do not iterate further to consider the effect of reflected transmission on the pumping power. This is justified as the overall reflection r observed in the samples is relatively weak ($< 10\%$). In this case, starting from the coupled-mode equations with nonlinear effects,

$$\frac{\mathrm{d}a_{\mathrm{res}}}{\mathrm{d}t} = -\left(\frac{\kappa_{\mathrm{res}}}{2} + i(\Delta_{\mathrm{res}} - (\alpha + g)\rho_{\mathrm{res}})\right) a_{\mathrm{res}} + \sqrt{\kappa_{e,\mathrm{res}}} a_{\mathrm{in}}, \tag{S39}$$

where $\rho_{\mathrm{res}} = |a_{\mathrm{res}}|^2/V_{\mathrm{eff}}$ is the resonance mode energy density, the transmission at steady state can be determined as

$$T_{\mathrm{res}} = 1 - \frac{\kappa_{e,\mathrm{res}}}{\kappa_{\mathrm{res}}/2 + i(\Delta_{\mathrm{res}} - (\alpha + g)\rho_{\mathrm{res}})}. \tag{S40}$$

The overall power transmission is approximated by substituting the above nonlinear T_{res} into Eq. (S38), and is used to fit the sum measurement data. The fitting results of the sum of Kerr and thermal coefficient $\alpha + g$ are shown in

Fig. 2b of main text.

B. Model of the ratio experiment

Here we derive Eq. (7)-(9) in the main text, which describe the response of the probe mode transmission when the power pumping the pump mode is modulated. The modulation can be broken down into three independent processes. The modulation of pumping power leads to the change of intracavity power in the pump mode, which leads to frequency shifts of the probe mode, which in turn leads to the transmission changes at a fixed frequency. The overall transfer function is the product of the three individual responses.

The dynamics of the pump mode reads

$$\frac{\mathrm{d}a_\mathrm{p}}{\mathrm{d}t} = -\frac{\kappa_\mathrm{p}}{2}a_\mathrm{p} + \sqrt{\kappa_{e,\mathrm{p}}}a_{\mathrm{in,p}}, \tag{S41}$$

where a_p is the pump mode amplitude, κ_p and $\kappa_{e,\mathrm{p}}$ are the total loss rate and external coupling rate of the pump mode, respectively, $a_{\mathrm{in,p}}$ is the pump input amplitude, and the frequency detuning term has been removed because the pump laser is locked close to the mode resonance in the experiment. The steady-state solution reads

$$a_\mathrm{p}^{(0)} = \frac{2}{\kappa_\mathrm{p}}\sqrt{\kappa_{e,\mathrm{p}}}a_{\mathrm{in,p}}^{(0)}, \tag{S42}$$

where $z^{(0)}$ is the steady-state value of variable z. Fourier transforming Eq. (S41) gives

$$i\Omega\widetilde{a_\mathrm{p}} = -\frac{\kappa_\mathrm{p}}{2}\widetilde{a_\mathrm{p}} + \sqrt{\kappa_{e,\mathrm{p}}}\widetilde{a_{\mathrm{in,p}}}, \quad \widetilde{a_\mathrm{p}} = \frac{1}{\kappa_\mathrm{p}/2 + i\Omega}\sqrt{\kappa_{e,\mathrm{p}}}\widetilde{a_{\mathrm{in,p}}}, \tag{S43}$$

where the AC component of z's Fourier transform is denoted as $\widetilde{z}$. Similarly

$$\widetilde{a_\mathrm{p}^*} = \frac{1}{\kappa_\mathrm{p}/2 + i\Omega}\sqrt{\kappa_{e,\mathrm{p}}}\widetilde{a_{\mathrm{in,p}}^*}. \tag{S44}$$

We denote $I_\mathrm{p} = |a_\mathrm{p}|^2$ as the intracavity energy and $P_\mathrm{in} = |a_{\mathrm{in,p}}|^2$ the pumping power. For small-signal modulations, we have

$$\widetilde{I_\mathrm{p}} \approx (a_\mathrm{p}^{(0)})^*\widetilde{a_\mathrm{p}} + a_\mathrm{p}^{(0)}\widetilde{a_\mathrm{p}^*}, \quad \widetilde{P_\mathrm{in}} \approx (a_{\mathrm{in,p}}^{(0)})^*\widetilde{a_{\mathrm{in,p}}} + a_{\mathrm{in,p}}^{(0)}\widetilde{a_{\mathrm{in,p}}^*}. \tag{S45}$$

After substituting $a_\mathrm{p}^{(0)}$ with $a_{\mathrm{in,p}}^{(0)}$ and using Eq. (S43) and (S44), we get

$$\frac{\widetilde{I_\mathrm{p}}(\Omega)}{\widetilde{P_\mathrm{in}}(\Omega)} = \frac{2\eta_\mathrm{p}}{i\Omega + \kappa_\mathrm{p}/2}, \tag{S46}$$

where $\eta_\mathrm{p} = \kappa_{e,\mathrm{p}}/\kappa_\mathrm{p}$ is the coupling efficiency for the pump mode. We note that the modulation of I_p also shifts the frequency of the pump mode. However, since I_p is maximized at zero detuning, such detuning changes do not influence I_p up to first order, which justifies dropping the detuning term from the beginning.

The resonance shift of the probe mode δ_b can be written similarly as Eq. (1) in the main text and reads

$$\frac{\widetilde{\delta_\mathrm{b}}(\Omega)}{\widetilde{I_\mathrm{p}}(\Omega)} = -\frac{\alpha\tilde{r} + \gamma g}{V_\mathrm{eff}}. \tag{S47}$$

Here $\widetilde{r}(\Omega)$ is the thermal response found in Section IC through simulation (with $\widetilde{r}(\Omega = 0) = 1$). The modulation frequency range is much lower than the electronic response in materials, thus we do not associate a response function to g. An extra correction factor γ is added to g as the probe mode sees cross-phase modulation rather than the previous self-phase modulation. For $Al_{0.2}Ga_{0.8}As$ and Ta_2O_5 resonators, a nearby mode in the same mode family is used as the probe mode, and in these cases $\gamma = 2$. For Si_3N_4 resonators, another transverse mode with a different polarization is used to improve contrast between thermal and Kerr effects, and the γ factor is evaluated to be $\gamma = 0.67$ (see Section IIIB).

Finally we calculate the transmission change of the probe mode $\widetilde{T_\mathrm{p}}$ with respect to $\widetilde{\delta_\mathrm{b}}$. The probe dynamics are

given by

$$\frac{\mathrm{d}a_\mathrm{b}}{\mathrm{d}t} = -\left(\frac{\kappa_\mathrm{b}}{2} + i\Delta_\mathrm{b}\right) a_\mathrm{b} + \sqrt{\kappa_{e,\mathrm{b}}}a_{\mathrm{in,b}}, \tag{S48}$$

where a_b is the probe mode amplitude, κ_b and $\kappa_{e,\mathrm{b}}$ are the total loss rate and external coupling rate of the probe mode, respectively, and $a_{\mathrm{in,b}}$ is the probe input amplitude. Its steady-state solution is

$$a_\mathrm{b}^{(0)} = \frac{\sqrt{\kappa_{e,\mathrm{b}}}a_{\mathrm{in,b}}}{\kappa_\mathrm{b}/2 + i\Delta_\mathrm{b}^{(0)}}, \tag{S49}$$

with $\Delta_\mathrm{b}^{(0)}$ the steady-state detuning of the probe mode.

Fourier transforming Eq. (S48) while expanding to first order of $\widetilde{\delta_\mathrm{b}}$ gives

$$i\Omega\widetilde{a_\mathrm{b}} = -(\frac{\kappa_\mathrm{b}}{2} + i\Delta_\mathrm{b})\widetilde{a_\mathrm{b}} - i\widetilde{\delta_\mathrm{b}}a_\mathrm{b}^{(0)}, \quad \widetilde{a_\mathrm{b}} = \frac{-ia_\mathrm{b}^{(0)}}{\kappa_\mathrm{b}/2 + i\Delta_\mathrm{b} + i\Omega}\widetilde{\delta_\mathrm{b}}, \tag{S50}$$

and $\widetilde{a_\mathrm{b}^*}$ can be found similarly. It should be noted that input probe amplitude $a_{\mathrm{in,b}}$ is not modulated and $\widetilde{a_{\mathrm{in,b}}}$ does not appear in Eq. (S50).

The transmitted power for the probe mode is found though the input-output relation:

$$T_\mathrm{b} = \left|a_{\mathrm{in,b}} - \sqrt{\kappa_{e,\mathrm{b}}}a_\mathrm{b}\right|^2. \tag{S51}$$

Because the probe input amplitude $a_{\mathrm{in,b}}$ is not modulated, $\widetilde{a_{\mathrm{in,b}}}$ doesn't show in the AC component of Eq. (S51). Fourier transforming Eq. (S51) while expanding to first order of $\widetilde{a_\mathrm{b}^*}$ gives

$$\widetilde{T_\mathrm{b}} = \left(a_{\mathrm{in,b}} - \sqrt{\kappa_{e,\mathrm{b}}}a_\mathrm{b}^{(0)}\right)\sqrt{\kappa_{e,\mathrm{b}}}\widetilde{a_\mathrm{b}^*} + \left[a_{\mathrm{in,b}}^* - \sqrt{\kappa_{e,\mathrm{b}}}\left(a_\mathrm{b}^{(0)}\right)^*\right]\sqrt{\kappa_{e,\mathrm{b}}}\widetilde{a_\mathrm{b}}. \tag{S52}$$

Plugging in $a_\mathrm{b}^{(0)}$ (Eq. (S49)) and $\widetilde{a_\mathrm{b}}$ (Eq. (S50)) results in

$$\widetilde{T_\mathrm{b}} = \frac{i\kappa_{e,\mathrm{b}}}{(\kappa_\mathrm{b}/2)^2 + \left(\Delta_\mathrm{b}^{(0)}\right)^2}\left[\frac{\kappa_\mathrm{b}/2 - \kappa_{e,\mathrm{b}} + i\Delta_\mathrm{b}^{(0)}}{\kappa_\mathrm{b}/2 + i\Omega - i\Delta_\mathrm{b}^{(0)}} - \frac{\kappa_\mathrm{b}/2 - \kappa_{e,\mathrm{b}} - i\Delta_\mathrm{b}^{(0)}}{\kappa_\mathrm{b}/2 + i\Omega + i\Delta_\mathrm{b}^{(0)}}\right]|a_{\mathrm{in,b}}|^2\,\widetilde{\delta_\mathrm{b}} \tag{S53}$$

$$= -\frac{2\kappa_{e,\mathrm{b}}\Delta_\mathrm{b}^{(0)}}{(\kappa_\mathrm{b}/2)^2 + \left(\Delta_\mathrm{b}^{(0)}\right)^2}\frac{\kappa_\mathrm{b} - \kappa_{e,\mathrm{b}} + i\Omega}{(\kappa_\mathrm{b}/2 + i\Omega)^2 + \left(\Delta_\mathrm{b}^{(0)}\right)^2}|a_{\mathrm{in,b}}|^2\,\widetilde{\delta_\mathrm{b}}. \tag{S54}$$

The overall response function is the product of responses of the three processes,

$$\widetilde{\mathcal{R}}(\Omega) = \frac{\widetilde{T_\mathrm{b}}(\Omega)}{\widetilde{P_\mathrm{in}}(\Omega)} = \frac{\widetilde{T_\mathrm{b}}(\Omega)}{\widetilde{\delta_\mathrm{b}}(\Omega)}\frac{\widetilde{\delta_\mathrm{b}}(\Omega)}{\widetilde{I_\mathrm{p}}(\Omega)}\frac{\widetilde{I_\mathrm{p}}(\Omega)}{\widetilde{P_\mathrm{in}}(\Omega)}, \tag{S55}$$

and corresponds to Eq. (9) in the main text. The last two factors (derived in Eq. (S47) and (S46)) correspond to Eq. (7) in the main text. The first factor (derived in Eq. (S54)) corresponds to Eq. (8) in the main text.

C. Sources of systematic errors in Q_abs and n_2

The Q_abs and n_2 are calculated from measured α and g factors, respectively, utilizing Eq. (2) in the main text. The calculation uses material constants from handbooks, where a measurement of these parameters is beyond the scope of this study.

- Thermal properties of the materials are taken from references as summarized in Table (S1) and (S2). The values not only depend upon the specific bulk material, but also on layer deposition and growth processes. In turn, a variation in these parameters will directly impact the inferred Q_abs. Using a larger thermal conductivity (or heat capacity) reduces the inferred $\widetilde{\delta T}$ for the same absorbed power (as in Eq. (S26)), and leads to a lower

calculated Q_{abs}. As an example, the reported thermal conductivity for Ta_2O_5 has variations and its impact is further discussed in Section IIID.

- Similarly, the optical refractive index n_o and group index n_g refer to bulk materials. These parameters appear in Eq. (S15) and affect the calculated n_2. Also, refractive indices determine the modal field distribution for averaging (as in Eq. (S11), (S13) and (S24)), which also affect the calculated material properties.
- In the experiment the air surrounding the sample is static, and the convective heat flux coefficient $h = 10$ W$\cdot$m$^{-2}\cdot$K^{-1} (ref.[16]) is used in all simulations. A larger convective heat flux coefficient will lead to lower temperature increase per unit absorbed power ($\overline{\delta T}/P_{\text{abs}}$), and to a lower calculated Q_{abs}. However, numerical simulations indicate that this effect is minor (see below).

$\overline{\delta T}/P_{\text{abs}}$ (K$\cdot$W^{-1})	SiO_2	Si_3N_4	$Al_{0.2}Ga_{0.8}As$	Ta_2O_5
$h = 2$ W$\cdot$m$^{-2}\cdot$K^{-1}	558	95.3	90.7	1085.1
$h = 10$ W$\cdot$m$^{-2}\cdot$K^{-1}	552	95.3	90.7	1085.1
$h = 25$ W$\cdot$m$^{-2}\cdot$K^{-1}	542	95.3	90.7	1085.1

Furthermore, there are physical processes neglected in the modelling that may lead to systematic errors.

- Surface and interface effects between different layers in the heterogeneous structure may alter the theoretical model. For example, contact thermal resistance is present at the interfaces between different materials, and this could not be evaluated here. However, for micron-scale cross-section structures in our study, the induced thermal resistance is negligible. As an example, for the interface between silicon and silica, numerical studies[17] and measurements[18] estimate the interface thermal resistance per unit area as 0.9×10^{-9} m$^2\cdot$K$\cdot$W^{-1} and 2.3×10^{-9} m$^2\cdot$K$\cdot$W^{-1}, respectively. Meanwhile, with parameters in Table. S1, the thermal resistance of a 1-micron-thick silica film is evaluated to be 7.1×10^{-7} m$^2\cdot$K$\cdot$W^{-1}, more than 10^2 larger than the interface contribution. Apart from contact thermal resistance, surface absorption may also be prominent for semiconductors ($Al_{0.2}Ga_{0.8}As$) and is discussed in Section IIIC.
- There are absorption pathways where a portion of the absorbed energy does not end up as heat (e.g., Raman and Brillouin scattering). Including such effects requires more absorbed power for the same $\overline{\delta T}$, and leads to a lower calculated Q_{abs}. We do not believe these processes are significant since the phase-matching condition is not favorable in measured devices.
- Harmonics generation, multi-photon absorption and other nonlinear optical effects may also lead to frequency shift or linewidth broadening. However, because these will induce a power-dependent loss on the pump mode, coupling efficiency of the pump mode will decrease when these effects are prominent, and this is not observed in the transmission traces (see Fig. S3d, S4d, S5d and S6d). As a result, these effects are also neglected in the model.

It should be noted that error bars in the main text represent measurement errors only (including instrumental error and data fitting error), while systematic errors discussed above are not included. To ensure accuracy, the mode used for testing should minimize all of the above nonlinear optical effects.

III. SUPPLEMENTARY NOTE 3: DETAILED MEASUREMENT RESULTS

A. Measurement of SiO_2 microresonators

The SiO_2 resonator is a disk with a wedge suspended in air, which supports a number of modes in each polarization. The fundamental TM mode (with the highest Q factor) is used for the measurements. The mode identification method is described elsewhere[19] and its profile is shown in Fig. S3a. To evaluate the mode temperature (as defined in Eq. (S18)) change upon optical absorption, i.e. $\overline{\delta T}/P_{\text{abs}}$ in Eq. 2 in the main text, finite-element method simulation is performed (Fig. S3a) using parameters in Section IC. The heating rate distribution is assumed to be proportional to the electric field intensity. Here, $\overline{\delta T}/P_{\text{abs}}$ is evaluated to be 552 K$\cdot$W^{-1}. Measured integrated mode dispersion $D_{\text{int}}/2\pi = (\omega_\mu - \omega_0 - \mu D_1)/2\pi$ of this mode family is shown in the Fig. S3b, along with a parabola fitting.

The simulated thermal diffusion responsivity $\tilde{r}(\Omega)$ (as defined in Eq. (S22)) is plotted in Fig. S3c using the method described in Section IC. The thermal relaxation bandwidth (3 dB) is <100 Hz, where the probe response is suppressed

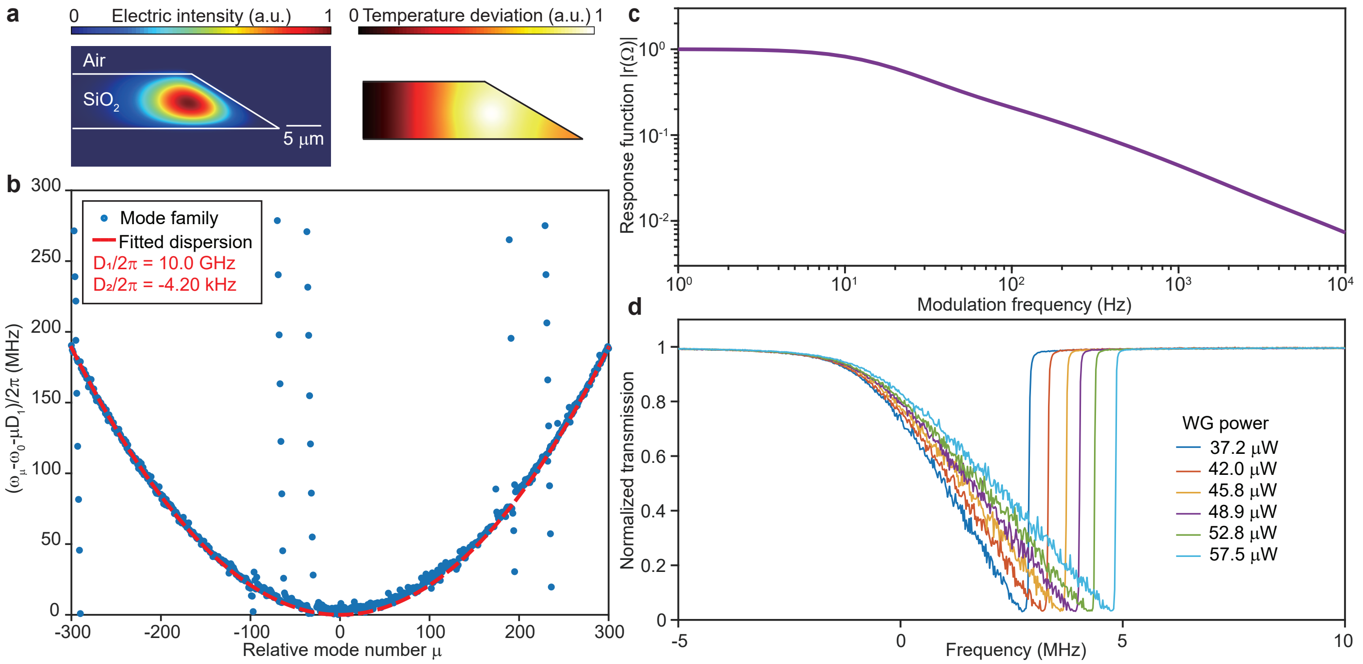

FIG. S3: **SiO_2 measurements. a,** Mode profiles of the measured mode family (left) and equilibrium temperature distribution of the resonator upon heating due to optical absorption (right). The geometry of the resonator is: radius 3.24 mm, thickness 8 μm, wedge angle 27° and undercut 137.5 μm. **b.** Measured dispersion spectrum of the experimental mode family. The dashed red line is the parabola fitting, and the dispersion parameters are fitted to be $D_1/2\pi = 10.0$ GHz and $D_2/2\pi = 4.20$ kHz. Relative mode number $\mu = 0$ corresponds to the wavelength of 1550 nm. **c.** Simulated thermal diffusion responsivity $r(\Omega)$ versus modulation frequency $\Omega/2\pi$. **d.** Representative normalized transmission spectra under different on-waveguide (WG) power. Intrinsic Q_0 and external (coupling) Q_e of this mode are 418.6 Million and 625.9 Million, respectively. This mode is at 1550nm.

by the servo feedback locking loop with a bandwidth of 1kHz (see Fig. 3b and 3c in the main text). Measuring the low-frequency photothermal response requires a locking bandwidth smaller than 10 Hz, which is challenging. Thus the ratio experiment is not performed on the silica resonator. Also, due to the suspended nature of the silica structure, the optical absorption effect for the current resonator is typically 10^2 higher than Kerr nonlinear effect ($g \ll \alpha$)[20]. Therefore, it is reasonable to neglect the Kerr contribution in the sum experiment, and attribute all the resonance shift to material absorption ($\alpha + g \approx \alpha$).

Representative linewidth broadening curves at different waveguide pumping power are plotted in Fig. S3d and exhibit the characteristic thermal 'triangle' features, as expected. Eq. (S38) (in this case r=0) and Eq. (S40) are used to fit the lineshape in Fig. S3d, and the thermal absorption coefficient α is extracted. As mentioned in Methods in the main text, the laser frequency must be tuned adiabatically to ensure thermal equilibrium at all times. This is checked by decreasing laser frequency scanning speed at constant power, while monitoring the transmission spectra, until the edge of the 'triangle' stops shifting to the red-detuned direction. Here the frequency scanning speed is set to 20 MHz s^{-1}, which is achieved by using a narrow-linewidth fiber laser.

This experiment is repeated using another SiO_2 resonator ($D_1/2\pi = 10$ GHz) with different mode polarization. The TE and TM modes are measured to have absorption Q factors of 4148 ± 264 M and 4160 ± 250 M, respectively. These results show that polarization does not significantly affect the measured absorption Q factors of the SiO_2 device. We note that spatial mode identification is not performed in this repeated experiment, which affects the value of $\overline{\delta T}/P_{\rm abs}$. For example, $\overline{\delta T}/P_{\rm abs}$ is evaluated to be 554, 537, 522 K·W^{-1} for TE0, TE1, TE2 modes and 552, 535, 519 K·W^{-1} for TM0, TM1, TM2 modes, respectively. As a result, the error uncertainty in the absorption Q factors for this device is larger.

B. Measurement of Si_3N_4 microresonators

The cross-section of the integrated Si_3N_4 resonator consists of a rectangular Si_3N_4 core with silica cladding at all sides. The resonator supports two transverse modes (fundamental TE and TM modes) and the fundamental TE mode

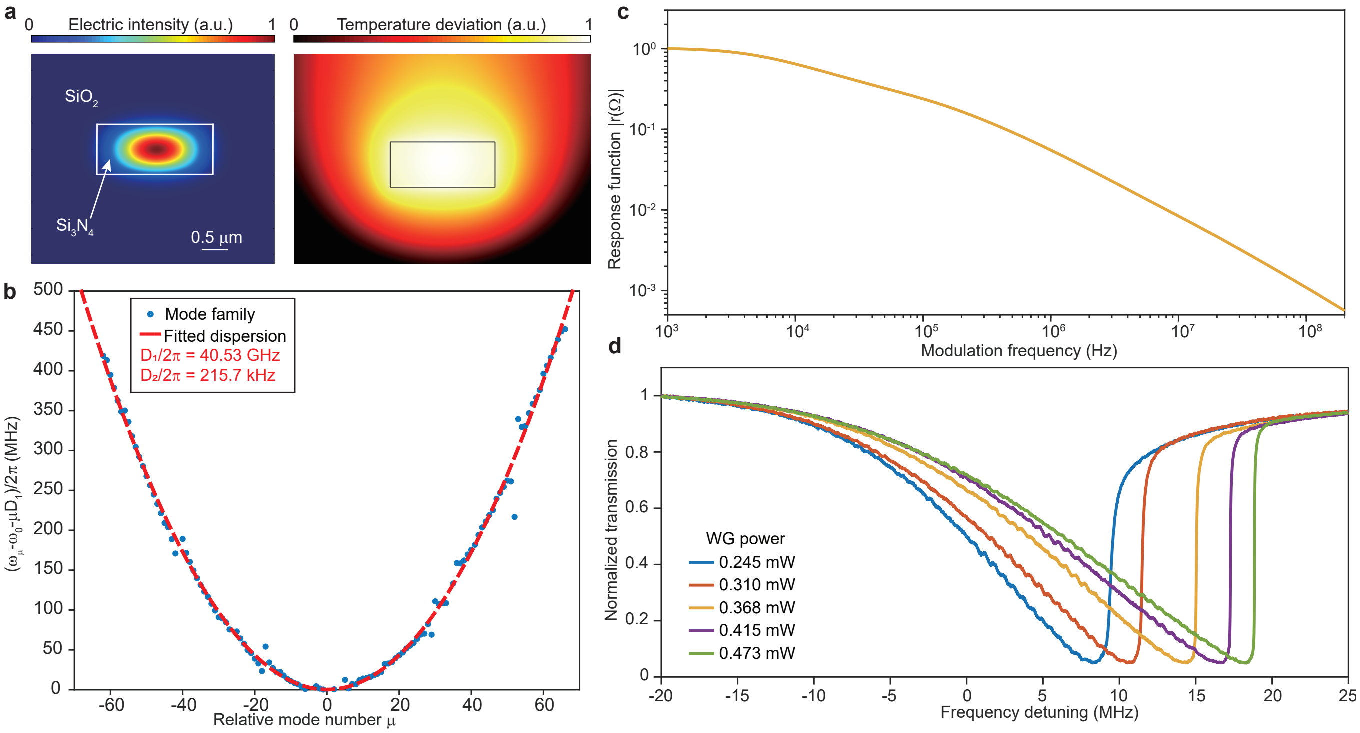

FIG. S4: **Si_3N_4 measurements. a,** Mode profile of the measured mode family (left) and temperature distribution in the resonator when heated by optical absorption (right). The Si_3N_4 core is 2.2 μm $\times$ 0.95 μm and is cladded by 3.45 μm-thick silica. The resonator radius is 0.562 mm. **b.** Measured dispersion spectrum of the measured mode family. The dashed red line is the parabola fitting with $D_1/2\pi = 40.53$ GHz and $D_2/2\pi = 215.7$ kHz. Relative mode number $\mu = 0$ corresponds to wavelength close to 1550 nm. **c.** Simulated thermal diffusion response $\tilde{r}(\Omega)$ versus modulation frequency $\Omega/2\pi$. **d.** Representative normalized transmission spectra under different on-waveguide (WG) power. Intrinsic Q_0 and external (coupling) Q_e of this mode are 29.0 Million and 52.1 Million, respectively. This mode is at 1544.1nm.

is used in sum measurement. The mode profile and temperature distribution upon optical absorption heating are shown in Fig. S4a. Here, $\overline{\delta T}/P_{\mathrm{abs}}$ is evaluated to be 95.3 K$\cdot$W^{-1}. The Dispersion spectrum is shown in Fig. S4b.

The thermal diffusion responsivity $\tilde{r}(\Omega)$ (as defined in Eq. (S22)) of this resonator is plotted in Fig. S4c with the technique described in Section IC. As an aside, thermal and Kerr effects are comparable in Si_3N_4, and in the ratio experiment the contrast between the two plateaus is low. In order to increase the contrast, pump and probe modes are chosen as two mode families with different polarizations (i.e. fundamental TE mode as pump, fundamental TM mode as probe). The cross phase modulation factor γ in this case is smaller than 2, which effectively suppresses the second (Kerr effect) plateau and increases the contrast between two plateaus. The cross phase modulation factor yields

$$\gamma = \frac{2\int n_o^2 n_2 \left[|\mathbf{F}_{\mathrm{TE}}|^2|\mathbf{F}_{\mathrm{TM}}|^2 + |\mathbf{F}_{\mathrm{TE}}\cdot\mathbf{F}_{\mathrm{TM}}^*|^2 + |\mathbf{F}_{\mathrm{TE}}\cdot\mathbf{F}_{\mathrm{TM}}|^2\right]\mathrm{d}V}{\int n_o^2 n_2\left[2|\mathbf{F}_{\mathrm{TE}}|^4 + |\mathbf{F}_{\mathrm{TE}}\cdot\mathbf{F}_{\mathrm{TE}}|^2\right]\mathrm{d}V}\frac{(\overline{n_o n_g})_{\mathrm{TE}}}{(\overline{n_o n_g})_{\mathrm{TM}}} = 0.67. \tag{S56}$$

In the sum experiment, the fundamnetal TE mode is tested at multiple wavelengths across the C-band. The result at 1544.1 nm under different pump power on waveguide is shown in Fig. S4d. A low scanning speed (12.7 GHz$\cdot$s^{-1}) is used to ensure the resonator reaches thermal equilibrium.

The correction factor for heterogeneous mode distribution in (S11), (S13) and (S24) is calculated based on finite-element method simulation, with

$$\overline{n_2} = 0.996 n_{2,\mathrm{Si_3N_4}} + 0.004 n_{2,\mathrm{SiO_2}}, \tag{S57}$$

$$\overline{n_o n_g} = 0.933 (n_o n_g)_{\mathrm{Si_3N_4}} + 0.067 (n_o n_g)_{\mathrm{SiO_2}}. \tag{S58}$$

$$\overline{\kappa_a} = 0.964\kappa_{a,\mathrm{Si_3N_4}} + 0.036\kappa_{a,\mathrm{SiO_2}}. \tag{S59}$$

These correction factors are applied to extract material absorption rate $\overline{\kappa_a}$ and nonlinearity $\overline{n_2}$. The n_o and n_g factors (at 1.55 μm) used for SiO_2 are $n_o = 1.44$, $n_g = 1.46$ (ref.[21]), for Si_3N_4 are $n_o = 2.00$, $n_g = 2.04$ (ref.[22]).

C. Measurement of $Al_{0.2}Ga_{0.8}As$ microresonators

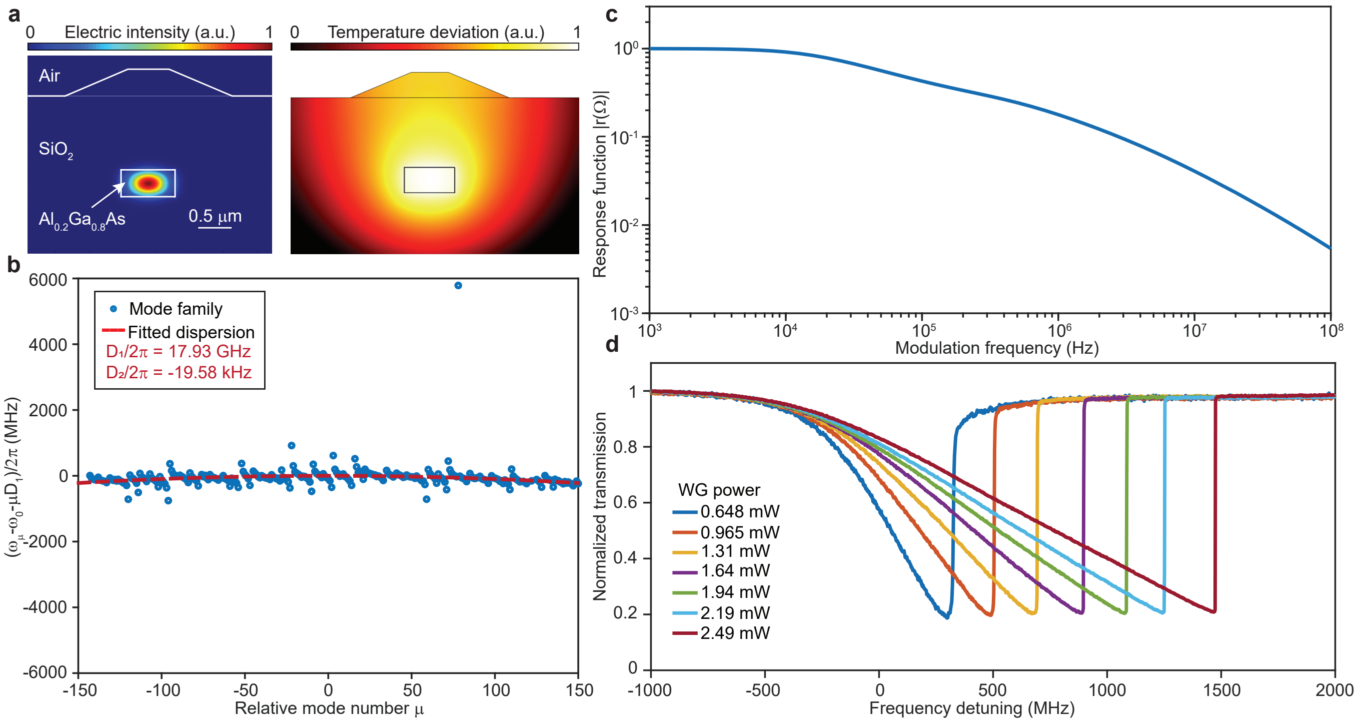

FIG. S5: **$Al_{0.2}Ga_{0.8}As$ measurements. a,** Mode profile of the measured mode family (left) and temperature distribution in the resonator upon optical absorption heating (right). The core is 0.8 μm $\times$0.4 μm $Al_{0.2}Ga_{0.8}As$ and is cladded by 1.5μm-thick silica. The resonator radius is 0.719 mm. It is noted that the top surface is not flat. **b.** Measured frequency dispersion of measured mode family. The dashed red line is parabola fitting with $D_1/2\pi = 17.93$ GHz and $D_2/2\pi = -19.58$ kHz. Relative mode number $\mu = 0$ corresponds to wavelength close to 1550 nm. **c.** Simulated thermal diffusion responsivity $\tilde{r}(\Omega)$ versus modulation frequency Ω. **d.** Representative normalized transmission spectra under different on-waveguide (WG) power. Intrinsic Q_0 and external (coupling) Q_e of this mode are 1.01 Million and 2.44 Million, respectively. This mode is at 1560.1nm.

The cross-section of the integrated $Al_{0.2}Ga_{0.8}As$ resonator consists of a rectangular $Al_{0.2}Ga_{0.8}As$ core with silica cladding at all sides. The cladding has protrusions on top of the resonator, as illustrated in Fig. S5a. The resonator supports two transverse modes (fundamental TE and TM modes) and the fundamental TE mode is used in both the sum and ratio measurements. The mode profile and equilibrium temperature distribution when heated by optical absorption from $Al_{0.2}Ga_{0.8}As$ core (i.e. bulk absorption) are shown in Fig. S5a. Here, $\overline{\delta T}/P_{\mathrm{abs}}$ is evaluated to be 90.7 K$\cdot$W^{-1}. Dispersion spectrum of this mode family is shown in Fig. S5b along with a parabola fitting with $D_1/2\pi = 17.93$ GHz and $D_2/2\pi = -19.58$ kHz.

We note that surface absorption may be prominent for aluminium gallium arsenide waveguides depending on fabrication details. This changes the distribution of absorbed heat for the waveguide cross-section. However, the $\overline{\delta T}/P_{\mathrm{abs}}$ coefficient from simulations differs by less than 1% even if the surface absorption is taken into account. This is because the spatially larger profile of the thermal distribution is not significantly affected by the exact location of the smaller modal heat source. The reported Q_{abs} here includes both bulk and surface contributions and, as noted in the main text, $Al_{0.2}Ga_{0.8}As$ resonators with Q factors higher than Q_{abs} have been reported elsewhere.

The thermal diffusion responsivity $\tilde{r}(\Omega)$ (as defined in Eq. (S22)) of the resonator is simulated using the finite-element method technique described in Section IC and is shown in S5c. In the ratio experiment the pump and probe modes belong to the same fundamental TE mode. In this case the cross phase modulation factor $\gamma = 2$.

In the sum experiment the fundamental TE mode is tested at multiple wavelengths across the C-band. The result at 1560.1 nm under different on-waveguide pumping power is shown in Fig. S5d. A low scanning speed (783.9 GHz·s^{-1}) is used to ensure that the resonator reaches thermal equilibrium. As an aside, two-photon absorption and harmonic generation will lead to a decrease in coupling efficiency as pump power increases[23]. Such effects are not significant in Fig. S5, indicating that the processes are not prominent for the current samples.

The correction factor for heterogeneous mode distribution in (S11) , (S13) and (S24) is calculated based on finite-element method,

$$\overline{n_2} = 0.997 n_{2,\mathrm{Al_{0.2}Ga_{0.8}As}} + 0.003 n_{2,\mathrm{SiO_2}}, \tag{S60}$$

$$\overline{n_o n_g} = 0.892 (n_o n_g)_{\mathrm{Al_{0.2}Ga_{0.8}As}} + 0.108 (n_o n_g)_{\mathrm{SiO_2}}. \tag{S61}$$

$$\overline{\kappa_\mathrm{a}} = 0.977 \kappa_{a,\mathrm{Al_{0.2}Ga_{0.8}As}} + 0.023 \kappa_{a,\mathrm{SiO_2}}. \tag{S62}$$

These correction factors are applied to extract material absorption rate $\overline{\kappa_\mathrm{a}}$ and nonlinearity $\overline{n_2}$. The n_o and n_g factors (at 1.55 μm) used for $Al_{0.2}Ga_{0.8}As$ are $n_o = 3.28$, $n_g = 3.22$ (ref.[24]).

D. Measurement of Ta_2O_5 microresonators

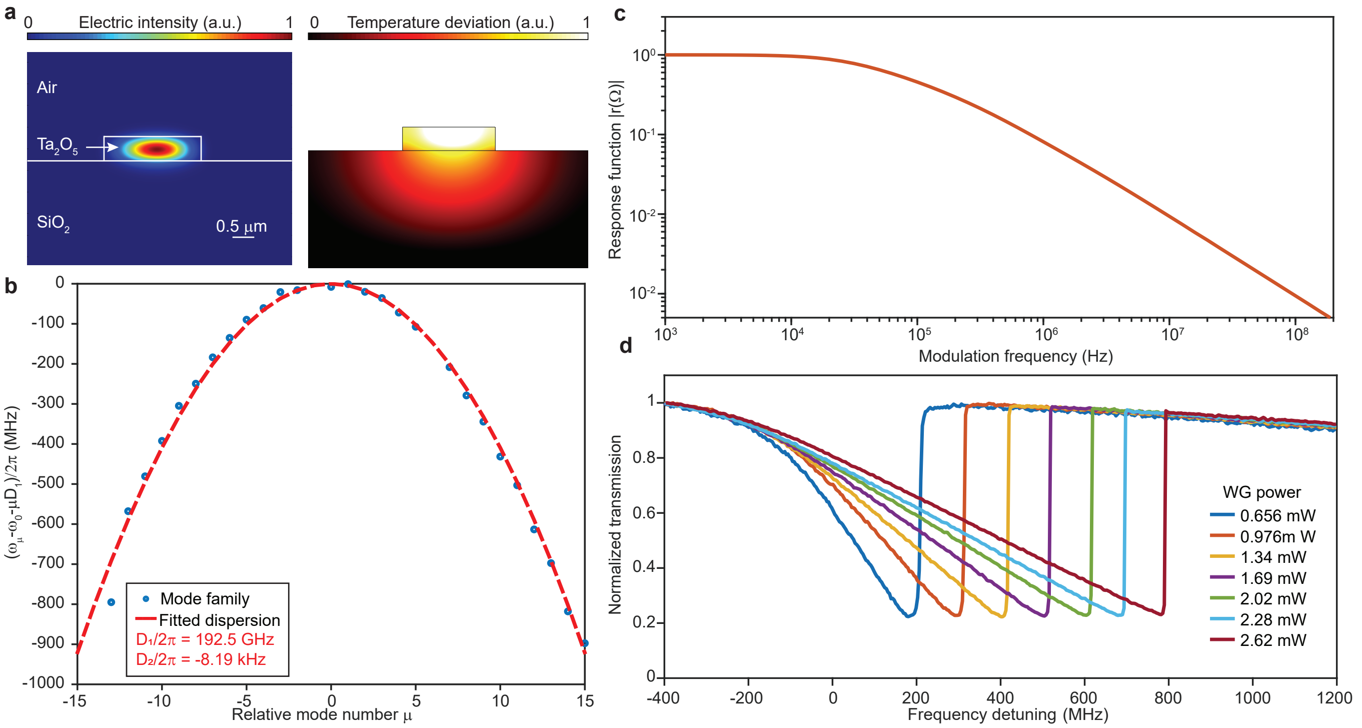

FIG. S6: **Ta_2O_5 measurements. a,** Mode profile of the measured mode family (left) and equilibrium temperature distribution inside the resonator upon optical absorption heating (right). The resonator consists a 2.25 μm × 0.57 μm core (Ta_2O_5) and a silica substrate. The resonator radius is 0.1095mm. **b.** Measured dispersion spectrum of the measured mode family. The dashed red line is parabola fit with $D_1/2\pi = 192.5$ GHz and $D_2/2\pi = -8.188$ MHz. Relative mode number $\mu = 0$ corresponds to wavelength close to 1550 nm. **c.** Simulated temperature response $\tilde{r}(\Omega)$ versus modulation frequency $\Omega/2\pi$. **d.** Representative normalized transmission spectra under different on-waveguide (WG) power. Intrinsic Q_0 and external (coupling) Q_e of this mode are 2.02 Million and 6.59 Million, respectively. This mode is around 1543.5nm.

The cross-section of the integrated Ta_2O_5 resonator consists of a rectangular Ta_2O_5 core on top of a silica substrate, and there's no cladding on the other sides of the resonator. The resonator supports two transverse modes (fundamental

TE and TM) and the fundamental TE mode is used in both the sum and ratio measurements. The mode profile and equilibrium temperature distribution when heated by optical absorption are shown in Fig. S6a. Here, $\overline{\delta T}/P_{\rm abs}$ is evaluated to be 1085.1 $K{\cdot}W^{-1}$ (using thermal conductivity 0.4 $W{\cdot}m^{-1}{\cdot}K^{-1}$). Dispersion spectrum is shown in Fig. S6b. The red dashed line indicates a parabola fitting with $D_1/2\pi = 195.2$ GHz and $D_2/2\pi = -8.188$ kHz.

In this study, the thermal conductivity assumed in the calculation of $Q_{\rm abs}$ was taken from that reported for electron-beam deposited Ta_2O_5, which is 0.4 $W{\cdot}m^{-1}{\cdot}K^{-1}$ (ref.[5]). Other reported values vary from 0.2 to 5 $W{\cdot}m^{-1}{\cdot}K^{-1}$ (ref.[25–27]) as thermal conductivity is strongly dependent upon deposition process among other factors. As discussed in section IIC, larger thermal conductivity will lead to faster thermal diffusion and therefore a lower value for $Q_{\rm abs}$ inferred from the measurements (see also Fig. S7). We also note that the measured value of Q_0 of the device places an upper bound on the total loss of the resonator. As absorption is only a component of the total resonator loss, Q_0 also therefore also sets a lower bound on $Q_{\rm abs}$.

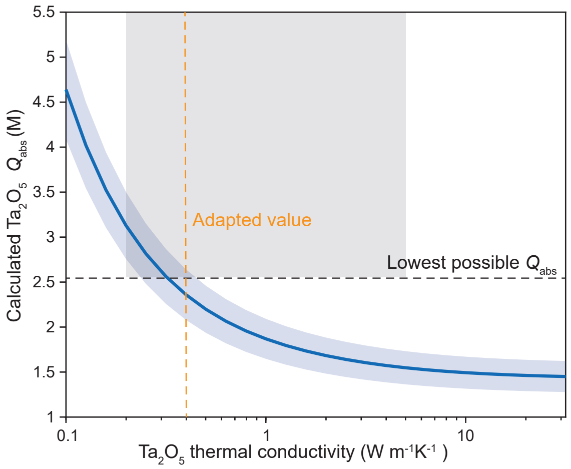

FIG. S7: **Calculated $Q_{\rm abs}$ of Ta_2O_5 versus thermal conductivity.** The calculated material limited $Q_{\rm abs}$ of Ta_2O_5 is plotted versus the thermal conductivity of Ta_2O_5 used in the FEM simulation. The light blue shading denotes the standard deviation from the measurements. The thermal conductivity value used in this study is marked with orange dashed line, while the reported range of values in the literature is shaded in gray. The lowest possible $Q_{\rm abs}$ as set by measured intrinsic Q factor is calculated from Eq. (S65) and is also plotted as the black dashed line.

The thermal diffusion responsivity $\tilde{r}(\Omega)$ (as defined in Eq. (S22)) of the resonator is simulated using the finite-element method technique described in Section IC and shown in S6c. In the ratio experiment the pump and probe modes belong to the same fundamental TE mode. In this case the cross phase modulation factor $\gamma = 2$.

In sum experiment the fundamental TE mode is tested at multiple wavelengths across the C-band. the result at 1543.5nm under different on-waveguide power is shown in Fig. S6d. A low scanning speed (1378 $GHz{\cdot}s^{-1}$) is used to ensure the thermal equilibrium.

The correction factor for heterogeneous mode distribution in (S11), (S13) and (S24) is calculated based on finite-element method,

$$\overline{n_2} = 0.989 n_{2,\rm Ta_2O_5} + 0.009 n_{2,\rm SiO_2} + 0.002 n_{2,\rm Air}, \tag{S63}$$

$$\overline{n_o n_g} = 0.874 (n_o n_g)_{\rm Ta_2O_5} + 0.080 (n_o n_g)_{\rm SiO_2} + 0.046 (n_o n_g)_{\rm Air}, \tag{S64}$$

$$\overline{\kappa_{\rm a}} = 0.946 \kappa_{a,\rm Ta_2O_5} + 0.042 \kappa_{a,\rm SiO_2} + 0.012 \kappa_{a,\rm Air}. \tag{S65}$$

These correction factors are applied to extract material absorption rate $\overline{\kappa_{\rm a}}$ and nonlinearity $\overline{n_2}$. The n_o and n_g factors (at 1.55 μm) used for Ta_2O_5 are $n_o = 2.06$, $n_g = 2.10$ (ref.[28]).

IV. SUPPLEMENTARY NOTE 4: MILLER'S RULE FOR NONLINEAR SUSCEPTIBILITY

The Miller's rule gives a phenomenological prediction of the material nonlinear coefficients based on its linear susceptibility[29,30]. Specifically, the third-order nonlinear susceptibility $\chi_{(3)}$ is predicted to be proportional to the

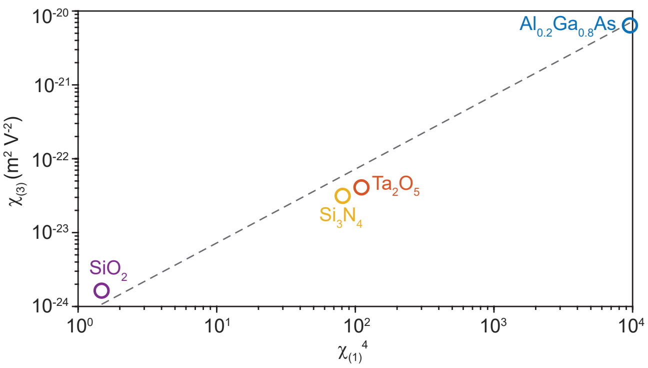

FIG. S8: **Observation of Miller's rule of nonlinear susceptibility.**

linear susceptibility $\chi_{(1)}^4$, where

$$\chi_{(3)} = \frac{4\varepsilon_0 c n_o^2}{3} n_2, \tag{S66}$$

$$\chi_{(1)} = n_o^2 - 1. \tag{S67}$$

The measured $\chi_{(3)}$ (as calculated from n_2) is plotted in Fig. S8, along with a fourth-power fitting (dashed). Despite a difference of 4 orders of magnitude in $\chi_{(3)}$ between different materials, the fourth-power scaling is evident as shown in the figure.

[1] Haynes, W. M. (ed.) *CRC Handbook of Chemistry and Physics* (CRC Press, 2016), 97th edn.
[2] Huang, G. *et al.* Thermorefractive noise in silicon-nitride microresonators. *Phys. Rev. A* **99**, 061801 (2019).
[3] Shur, M. S. *Handbook Series on Semiconductor Parameters - Volume 2: Ternary and Quaternary III-V Compounds*, 1–36 (World Scientific Publishing Company, Singapore, 1999).
[4] Martin, P. *et al.* Mechanical and Optical Properties of The Films of Tantalum Oxide Deposited by Ion-Assisted Deposition. *Mater. Res. Soc. Symp. Proc.* **308**, 583 (1993).
[5] Farsi, A., Siciliani de Cumis, M., Marino, F. & Marin, F. Photothermal and thermo-refractive effects in high reflectivity mirrors at room and cryogenic temperature. *J. Appl. Phys.* **111**, 043101 (2012).
[6] Samsonov, G. V. *The Oxide Handbook*, 145. IFI Data Base Library (Springer US, 1973).
[7] Gao, H. *et al.* Investigation on the thermo-optic coefficient of silica fiber within a wide temperature range. *J. Light. Technol.* **36**, 5881–5886 (2018).
[8] Elshaari, A. W., Zadeh, I. E., Jöns, K. D. & Zwiller, V. Thermo-Optic Characterization of Silicon Nitride Resonators for Cryogenic Photonic Circuits. *IEEE Photon. J.* **8**, 10 (2016).
[9] Chang, L. *et al.* Ultra-efficient frequency comb generation in algaas-on-insulator microresonators. *Nat. Commun.* **11**, 1–8 (2020).
[10] Wu, C.-L. *et al.* Tantalum pentoxide (Ta_2O_5) based athermal micro-ring resonator. *OSA Continuum* **2**, 1198–1206 (2019).
[11] Kim, J. P. & Sarangan, A. M. Temperature-dependent Sellmeier equation for the refractive index of $Al_xGa_{1-x}As$. *Opt. Mater. Express* **8**, 485–490 (2018).
[12] Matsko, A. B., Savchenkov, A. A., Yu, N. & Maleki, L. Whispering-gallery-mode resonators as frequency references. i. fundamental limitations. *J. Opt. Soc. Am. B* **24**, 1324–1335 (2007).
[13] Tien, C.-L. & Lin, T.-W. Thermal expansion coefficient and thermomechanical properties of SiN_x thin films prepared by plasma-enhanced chemical vapor deposition. *Appl. Opt.* **51**, 7229–7235 (2012).
[14] Tien, C.-L., Lee, C.-C., Chuang, K.-P. & Jaing, C.-C. Simultaneous determination of the thermal expansion coefficient and the elastic modulus of Ta_2O_5 thin film using phase shifting interferometry. *J. Mod. Opt.* (2009).
[15] Okada, Y. & Tokumaru, Y. Precise determination of lattice parameter and thermal expansion coefficient of silicon between 300 and 1500 K. *J. Appl. Phys.* **56**, 314–320 (1984).
[16] Çengel, Y. A. *Heat Transfer: A Practical Approach* (McGraw-Hill, 2003).
[17] Chen, J., Zhang, G. & Li, B. Thermal contact resistance across nanoscale silicon dioxide and silicon interface. *J. of Appl. Phys.* **112**, 064319 (2012).
[18] Hurley, D. H., Khafizov, M. & Shinde, S. Measurement of the kapitza resistance across a bicrystal interface. *J. of Appl.*

Phys. **109**, 083504 (2011).
[19] Yi, X. *et al.* Single-mode dispersive waves and soliton microcomb dynamics. *Nat. Commun.* **8**, 14869 (2017).
[20] Rokhsari, H. & Vahala, K. J. Observation of kerr nonlinearity in microcavities at room temperature. *Opt. Lett.* **30**, 427–429 (2005).
[21] Malitson, I. H. Interspecimen Comparison of the Refractive Index of Fused Silica. *J. Opt. Soc. Am.* **55**, 1205–1209 (1965).
[22] Luke, K., Okawachi, Y., Lamont, M. R. E., Gaeta, A. L. & Lipson, M. Broadband mid-infrared frequency comb generation in a Si_3N_4 microresonator. *Opt. Lett.* **40**, 4823–4826 (2015).
[23] Parrain, D. *et al.* Origin of optical losses in gallium arsenide disk whispering gallery resonators. *Opt. Express* **23**, 19656–19672 (2015).
[24] Papatryfonos, K. *et al.* Refractive indices of MBE-grown $Al_xGa_{1-x}As$ ternary alloys in the transparent wavelength region. *AIP Adv.* **11**, 025327 (2021).
[25] Wu, Z. L., Reichling, M., Hu, X.-Q., Balasubramanian, K. & Guenther, K. H. Absorption and thermal conductivity of oxide thin films measured by photothermal displacement and reflectance methods. *Appl. Opt.* **32**, 5660 (1993).
[26] Landon, C. D. *et al.* Thermal transport in tantalum oxide films for memristive applications. *Appl. Phys. Lett.* **107**, 023108 (2015).
[27] Grilli, M., Ristau, D., Dieckmann, M. & Willamowski, U. Thermal conductivity of e-beam coatings. *Appl. Phys. A* **71**, 71–76 (2000).
[28] Bright, T. J. *et al.* Infrared optical properties of amorphous and nanocrystalline Ta_2O_5 thin films. *J. Appl. Phys.* **114**, 083515 (2013).
[29] Miller, R. C. Optical second harmonic generation in piezoelectric crystals. *Appl. Phys. Lett.* **5**, 17–19 (1964).
[30] Ettoumi, W., Petit, Y., Kasparian, J. & Wolf, J.-P. Generalized miller formulæ. *Opt. Express* **18**, 6613–6620 (2010).
